# Anaphylactic shock due to traumatic rupture of pulmonary hydatid cyst: Case report

**DOI:** 10.1016/j.ijscr.2021.105660

**Published:** 2021-03-03

**Authors:** Aabdi Mohammed, Khalil Malki, Yassine Mellagui, Houssam Bkiyar, Brahim Housni

**Affiliations:** Anesthesiology and Intensive Care Unit Department, Faculty of Medicine and Pharmacy of Oujda, Mohammed VI University Hospital, Mohammed I University, Oujda, Morocco

**Keywords:** Hydatid cyst, Anaphylactic shock, Traumatic, Rupture

## Abstract

**Introduction:**

Hydatid cyst is an infectious disease caused by « ECHINOCOCCUS GRANULOSIS », it can be asymptomatic or manifests depending on size, location, and relation with other organs, or by complication like rupture. This latter might occur spontaneously or post-traumatic, and it might manifest with an anaphylactic shock, a life-threatening situation.

**Importance:**

Anaphylactic shock is a rare cause of traumatic shock state.

**Case presentation:**

A 30 years old man with no medical history was admitted to the emergency room after a car accident, on his admission, his Glasgow coma scale was 10/15 with a blood pressure of 80/30 mmHg, he was intubated and stabilized hemodynamically. The full-body CT scan showed no abnormalities except on the thoracic level where it showed a well-limited rounded formation with regular contours containing hydro-aeric level related to ruptured hydatid cyst.

After ruling out the diagnosis of hemorrhagic, hypovolemic shock, the diagnosis of anaphylactic shock due to a post-traumatic rupture of the hydatid cyst was maintained.

**Conclusion:**

Post-traumatic rupture of the hydatid cyst is a rare emergency that requires early diagnosis and management. Surgery remains the principal treatment of ruptured hydatid cyst.

Anaphylactic shock is a life-threatening situation with various symptoms; epinephrine is the cornerstone for management of this type of shock.

## Highlights

• Hydatid cyst can be asymtpmatic or manifesrs depending on size, location with other organs, or by complication like rupture.
• This latter might occur spontaneously or post-traula, and it might manifests with an anaphylactic shock, a life-threatening situation.
• Anaphylactic shock is a rare etiology of post traumatic shock state.

## 1 Introduction

Hydatid cyst is caused by « ECHINOCOCCUS GRANULOSUS», it remains a serious health problem in many countries [1]. It is usually asymptomatic with accidental discovery, clinical manifestations depend on size, location, and its relation with other structures, and it is possible to be revealed by complications like rupture, infection, anaphylactic shock, or compression [[2], [3], [4]].

In this paper, we will represent a rare case of a 30 years old man victim of a car accident leading to a rupture of an unknown pulmonary hydatid cyst manifesting with an anaphylactic shock.

## 2 Case presentation

A previously healthy 30 years old man, with no medical history, was admitted to the emergency room after a car accident: rollover by his car.

The physical examination on his admission was as follows: hearts rate of 145 beats/minute, blood pressure of 80/30 mmHg, with no signs of external bleeding, medullar trauma **or** limb fracture, the respiratory rate was 25 cycles/min, and pulse oximetry 75% on ambient air, 85% on high concentration mask with ronchis on the right side. The Glasgow Coma Scale GCS was 10/15 (eyes opening response 2/4, verbal response 3/5, and motor response 4/5) with contracted pupils, and no localization signs.

At this moment a decision to put the patient on mechanical ventilation was taking with inspiratory fraction of oxygen at 100%, tidal volume og 6 mL/kg and pression end expiratory pressure at 3mBar, with pulse oximetry at 93%.

The complete blood count showed the following: hemoglobin 12 g/dl, platelets 350000/mL, **prothrombin time** 80%, fibrinogen 3.5 g/l, white blood cells 7500/mL, eosinphils 55%, normal renal function : **creatinine 0.8 mg/dl,** urea 0.30 g/L.

**Fig. 1:**
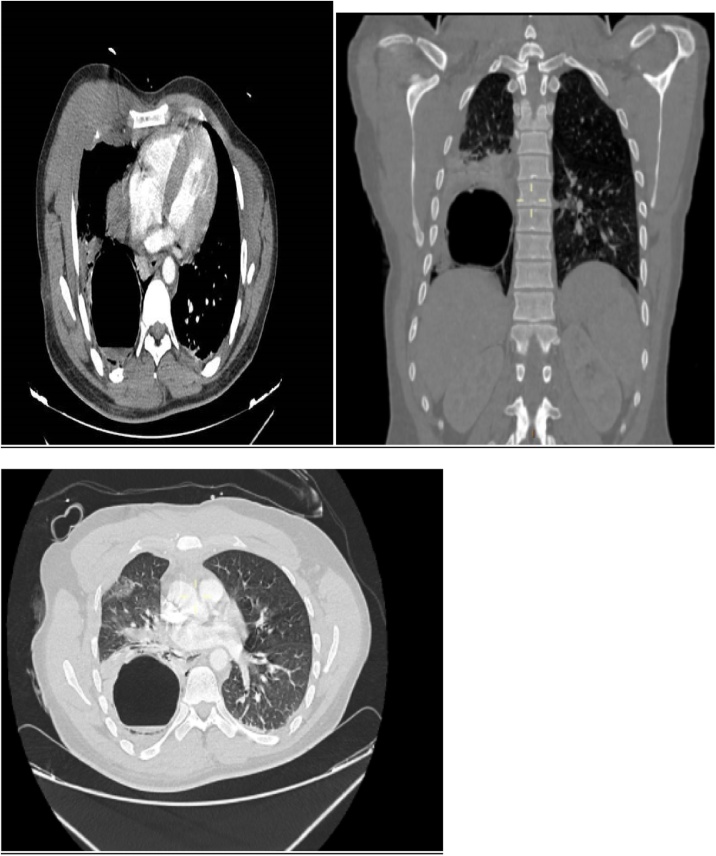
CT SCAN showing well limited rounded formation of regular contours containing a hydro-aeric level related to a ruptured hydatid cyst.

After hemodynamic stabilization by fluid restitution with 500 mL of saline serum, and introduction of norepinephrine 1 mg/h, the full-body computerized tomography CT scan was performed, and it showed no internal bleeding, pneumothorax or medullar trauma, and showed on the thoracic level where it showed a well limited rounded formation of regular contours containing a hydro-aeric level related to a ruptured hydatid cyst (Fig. 1).

After ruling out the possibility of a hemorrhagic, hypovolemic shock, the diagnosis of anaphylactic shock due to post-traumatic rupture of hydatid cyst was maintained, norepinephrine was switched with epinephrine with a dose of 2 mg/h, and we gave him 500 mg of hydrocortisone with improvement in the hemodynamic state.

**Fig. 2:**
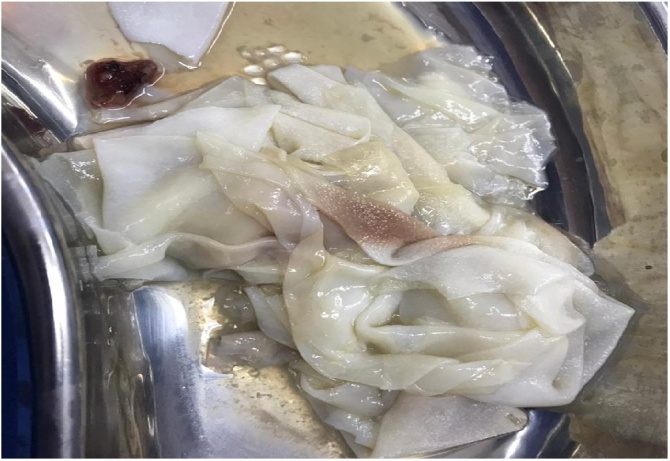
Ruptured specimen of hydadit cyst of lung.

The patient was admitted to the operating room the next day for cyst removal by open thoracotomy (Fig. 2).

In the postoperative period, Albendazole was started with a dose of 400 mg/day.

The evolution was favorable; he was extubated in the immediate post-operative period and referred to the thoracic department.

## 3 Discussion

Hydatid cyst is a zoonotic infectious disease caused by « ECHINOCOCCUS GRANULOSUS », it is transmitted to human, an intermediated host, by direct contact with dogs or sheeps [[5], [6], [7]].

The lung is the second frequent localization of hydatid cyst after liver infection [8,9].

Because of the delay of the diagnosis, the hydatid cyst remains asymptomatic for years and grows to big sizes and the clinical signs depend generally on the location, size, and relation with adjacent organs [2,10,11]. Its severity is due to complication like infection or rupture; these complications can even cause death in severe cases [7,12]

The rupture of the hydatid cyst might occur spontaneously due to increased pressure or size of the cyst or post-traumatic, the latter is a rare event with high morbidity and lethality rates [6,7].

The clinical signs of ruptured pulmonary hydatid cyst might include coughing, chest pain, hemoptysis, hydatidemesis, and anaphylactic shock, a life-threatening situation [[13], [14], [15]].

CT scan is the gold standard for hydatic cyst diagnostic with a sensitivity of 95%, the images of ruptured hydatic cyst show essentially hydro-aeric levels [16,17].

The diagnosis of ruptured hydatid cyst must be immediate because it requires appropriate surgical management [7].

Anaphylactic shock might be revealed with cardiovascular signs (collapse…), respiratory signs (wheezing, dyspnea…), dermatologic signs (flushing, urticaria, angioedema…), and neurological signs (seizures, headache…) [18]. **Cutaneous and mucosal signs are often the first signs to appear but they might be missing, especially during severe reactions with immediate state of shock** [19]. Epinephrine is the cornerstone and it must be started immediately one the diagnosis of anaphylactic shock is made, other pharmacotherapies are considered: antihistaminic, steroids, and quick fluid replacement [18].

Surgery remains the principal treatment of ruptured hydatid cyst, the mean goal of surgery is to eliminate the cyst, minimize morbidity and reduce recurrences [20].

The principal of surgery consists on complete excision with maximum preservation of lung tissue [5].

Albendazole, with a dose of 10 mg/kg/day for 12 months should be started on the immediate post-operative period to avoid recurrences [7].

## 4 Conclusion

Post-traumatic rupture of pulmonary hydatid cyst is a rare life-threatening situation requiring an early diagnosis and proper management to avoid severe complications that might include death.

CT scan is the gold standard to make the diagnosis of ruptured hydatid cyst and surgery remains the essential treatment.

The work has been reported in line with the SCARE 2020 guideline [21].

## Declaration of Competing Interest

The authors state that they have no conflicts of interest for this report.

## Funding

This research did not receive any specific grant from funding agencies in the public, commercial, or not-for-profit sectors.

## Ethical approval

The ethical committee approval was not required given the article type (case report).

## Consent

Written informed consent was obtained from the patient for publication of this case report and accompanying images.

## Author contribution

AABDI Mohammed: study concept, Data collection; data analysis; writing review & editing.

MALKI Khalil: Study conception, data analysis.

MELLAGUI Yassine: contributor.

BKIYAR Houssam: Supervision and data validation.

HOUSNI Brahim: supervision and data validation.

## Registration of research studies

Not Applicable.

## Guarantor

AABDI Mohammed.

MALKI Khalil.

## Provenance and peer review

Not commissioned, externally peer-reviewed.

## Availability of data and materials

The data used to support the findings of this study are available from the corresponding author upon request.

## CRediT authorship contribution statement

**Aabdi Mohammed:** Writing - original draft, Conceptualization, Methodology, Software, Validation, Formal analysis, Visualization. **Khalil Malki:** Resources, Data curation, Software. **Yassine Mellagui:** Formal analysis, Visualization. **Houssam Bkiyar:** Writing - review & editing, Visualization. **Brahim Housni:** Project administration, Visualization, Writing - review & editing, Resources, Conceptualization, Methodology, Validation.
